# Supplementary material for: The Small RNA Universe of Capitella teleta
Source: Front Mol Biosci. 2022 Feb 25;9:802814. doi: 10.3389/fmolb.2022.802814 (PMC8915122; doi:10.3389/fmolb.2022.802814)
Supplement: Supplementary file 1 [file DataSheet1.ZIP › Supplement/known/CAPTEscaffold_481_21028.pdf]

Provisional ID	:	CAPTEscaffold_481_21028
Score total	:	3864.1
Score for star read(s)	:	3.9
Score for read counts	:	3854.4
Score for mfe	:	1.2
Score for randfold	:	1.6
Score for cons. seed	:	3
Total read count	:	7572
Mature read count	:	5077
Loop read count	:	0
Star read count	:	2495

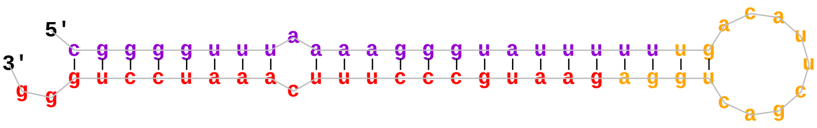

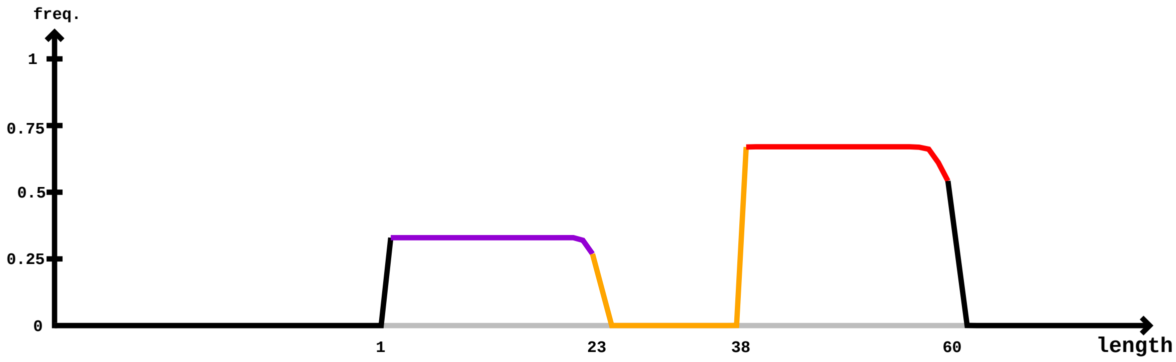


```
                                      Star                                 Mature
5'- ucuucuucuucuucuucacccaucagauauauucgggguuuaaaaggguauuuuuugacauucgacuggagaaugcccuuucaaauccugggucucucugagccugacaucu  -3'  obs
    ucuucuucuucuucuucacccaucagauauauucgggguuuaaaaggguauuuuuugacauucgacuggagaaugcccuuucaaauccugggucucucugagccugacaucu       exp
    ucuucuucuucuucuucacccaucagauauauucgggguuuaaaaggguauuuuuugacauucgacuggagaaugcccuuucaaauccugggucucucugagccugacaucu       known
    ...............(((....(((((...(((((((((((.(((((((((((((((.........))))))))))))))).)))))))))))...)))))...))).....   reads   mm   sample

    .................................cAggguuuaaaaggguauuu...........................................................   1       1    seq
    .................................cgggguuuaaaaggguauuu...........................................................   73      0    seq
    .................................cgggguuuaaaagAguauuuu..........................................................   1       1    seq
    .................................cgggguuuaaaaggguauuuu..........................................................   385     0    seq
    .................................cgggguuAaaaaggguauuuuu.........................................................   1       1    seq
    .................................Ngggguuuaaaaggguauuuuu.........................................................   3       1    seq
    .................................cgggguuuaaaaggguauGuuu.........................................................   1       1    seq
    .................................cggggAuuaaaaggguauuuuu.........................................................   1       1    seq
    .................................cgggguuuaaaaggguauuGuu.........................................................   1       1    seq
    .................................cgggguuuaaaaggguauuuuA.........................................................   1       1    seq
    .................................cgggguuuaaaaAgguauuuuu.........................................................   1       1    seq
    .................................cgggguuuaaaaggguauuuuu.........................................................   1959    0    seq
    .................................cgAgguuuaaaaggguauuuuu.........................................................   5       1    seq
    .................................cgggguuuaaaaggguaGuuuu.........................................................   1       1    seq
    .................................cgggguuuGaaaggguauuuuu.........................................................   54      1    seq
    .................................cgggguuuaaaagggAauuuuu.........................................................   2       1    seq
    .................................cUggguuuaaaaggguauuuuu.........................................................   1       1    seq
    .................................Agggguuuaaaaggguauuuuu.........................................................   1       1    seq
    .................................cgggguAuaaaaggguauuuuu.........................................................   2       1    seq
    .................................cgggguuuaaaaggguauuuuuu........................................................   1       0    seq
    ...................................................uuuuugacauucgacugga..........................................   1       0    seq
    .....................................................................agaaugcccuuucaaauccug......................   1       0    seq
    .....................................................................agaaugcccuuucaaauccugg.....................   1       0    seq
    ......................................................................gaaugcccuuucaaaucc........................   12      0    seq
    ......................................................................gaaugcccuuucaaauccG.......................   1       1    seq
    ......................................................................gaaugcccuuucaaauccu.......................   54      0    seq
    ......................................................................gaaugcccuuuAaaauccu.......................   1       1    seq
    ......................................................................gaaugcccuuucaaaucUug......................   1       1    seq
    ......................................................................gGaugcccuuucaaauccug......................   1       1    seq
    ......................................................................gaauAcccuuucaaauccug......................   1       1    seq
    ......................................................................gaaugcccuuucaaaCccug......................   1       1    seq
```

```
                                 Star                                Mature
ucuucuucuucuucuucacccaucagauauauucgggguuuaaaaggguauuuuuugacauucgacuggagaaugcccuuucaaauccugggucucucugagccugacaucu
......................................................................gaaugcccuuucaaauccGg......................      2     1     seq
......................................................................gaaugcccuuuUaaauccug......................      1     1     seq
......................................................................Aaaugcccuuucaaauccug......................      1     1     seq
......................................................................gaaugcAcuuucaaauccug......................      1     1     seq
......................................................................gaaugcccuuucaaauccug......................      368   0     seq
......................................................................gaaugcccuuuAaaauccug......................      2     1     seq
......................................................................gaaugcccuuAcaaauccug......................      1     1     seq
......................................................................gaauAcccuuucaaauccugg.....................      2     1     seq
......................................................................gaaugcccuuucaaauccugg.....................      504   0     seq
......................................................................gaaugUccuuucaaauccugg.....................      1     1     seq
......................................................................gaaugccUuuucaaauccugg.....................      1     1     seq
......................................................................gaaugccAuuucaaauccugg.....................      1     1     seq
......................................................................gaaugcccuuucaaauccAgg.....................      1     1     seq
......................................................................Naaugcccuuucaaauccugg.....................      1     1     seq
......................................................................gaaugcccuuucaaauccugU.....................      1     1     seq
......................................................................Caaugcccuuucaaauccugg.....................      1     1     seq
......................................................................gaaugcccuuucaaaucUugg.....................      1     1     seq
......................................................................gaaugcccuuucaaauccuAg.....................      1     1     seq
......................................................................Aaaugcccuuucaaauccugg.....................      4     1     seq
......................................................................gaaugcccuuucGaauccugg.....................      1     1     seq
......................................................................gaaugcccCuucaaauccuggg....................      1     1     seq
......................................................................Naaugcccuuucaaauccuggg....................      1     1     seq
......................................................................gaaugcccuuucaaauccuAgg....................      23    1     seq
......................................................................gaaugccUuuucaaauccuggg....................      4     1     seq
......................................................................gaaugcccuuuUaaauccuggg....................      5     1     seq
......................................................................gaaugcccuuucaaaucUuggg....................      2     1     seq
......................................................................gaauUcccuuucaaauccuggg....................      1     1     seq
......................................................................gaaugcccuuucaaauccuggU....................      26    1     seq
......................................................................gaaugcccuAucaaauccuggg....................      3     1     seq
......................................................................gaaugcccuuuGaaauccuggg....................      1     1     seq
......................................................................gaaugcGcuuucaaauccuggg....................      2     1     seq
......................................................................Aaaugcccuuucaaauccuggg....................      22    1     seq
......................................................................gaaugcccuuucaaauccugCg....................      1     1     seq
......................................................................gaaugcccuuucaaauccuggg....................      3920  0     seq
......................................................................Caaugcccuuucaaauccuggg....................      1     1     seq
......................................................................gaaugcccuuucaaauccuggA....................      3     1     seq
......................................................................gaaugcccuuucaaauccugAg....................      1     1     seq
......................................................................gaaugcccuuucaaauccuggC....................      3     1     seq
......................................................................gaaugcccuuucaaaAccuggg....................      3     1     seq
......................................................................gaaugccGuuucaaauccuggg....................      1     1     seq
......................................................................gaaugcAcuuucaaauccuggg....................      6     1     seq
......................................................................gaaugcccuuucaaaucGuggg....................      1     1     seq
......................................................................gaGugcccuuucaaauccuggg....................      1     1     seq
......................................................................gaaugcccuuuAaaauccuggg....................      4     1     seq
......................................................................gaaugcccuuucaaaucAuggg....................      1     1     seq
......................................................................gaaugcccuuAcaaauccuggg....................      3     1     seq
......................................................................gaaugAccuuucaaauccuggg....................      3     1     seq
......................................................................Uaaugcccuuucaaauccuggg....................      2     1     seq
......................................................................gaaAgcccuuucaaauccuggg....................      1     1     seq
......................................................................gaaugcccuuucaaauccugUg....................      2     1     seq
......................................................................gaaugcccuuucaaauccuUgg....................      1     1     seq
......................................................................gaaugcccuuucaaGuccuggg....................      1     1     seq
......................................................................gaaugcccuuucGaauccuggg....................      4     1     seq
......................................................................gaUugcccuuucaaauccuggg....................      1     1     seq
......................................................................gaaugcccuuucaaauccugggG...................      19    1     seq
......................................................................gaaugcccuuucaaauccugggu...................      5     0     seq
......................................................................gaaugcccuuucaaauccuggUu...................      4     1     seq
......................................................................gaaugcccuuucaaauccugggA...................      16    1     seq
......................................................................Aaaugcccuuucaaauccugggu...................      1     1     seq
.......................................................................aaugcccuuucaaauccuggg....................      2     0     seq
.......................................................................aaugcccuuucaaauccugggA...................      1     1     seq
.......................................................................aaugcccuuucaaauccuggguU..................      4     1     seq
```
